# An Inquiry, Critical and Experimental, Etc.

**Published:** 1853-08

**Authors:** N. S. Davis

**Affiliations:** Professor of Pathology, Practice of Medicine, and Clinical Medicine, in Rush Medical College; and one of the Physicians to the Ill. Gen. Hospital


*An Inquiry, Critical and Experimental, into the Pathology of Fever.* By N. S. DAVIS, M. D. Professor of Pathology, Practice of Medicine, and Clinical Medicine, in Rush Medical College; and one of the Physicians to the Ill. Gen. Hospital.

[CONTINUED FROM PAGE 176.]

AGAIN, if the same original cause be generated and introduced into the system with its properties modified in the opposite direction, it so changes the relations of the blood to the solids that the vital or molecular affinities are suspended, and of course the primary functions are suspended also, constituting a stage of depression or chill, with scarcely an available element of reaction. And this constitutes the pernicious or malignant intermittent. Such cases are often regarded as congestive, but they differ just as much from proper congestion, as do the phenomena that precede and accompany an ordinary chill differ from those of "simple nervous depression." The three cases here supposed are intended to represent the three well known varieties of periodical or malarious fever. Between the active remittent and the well marked pernicious intermittent there is truly a wide difference. The one is characterized by a high irritative fever continuing with remissions only for two or three weeks, unless cut short by appropriate remedies, while the other exhibits the very opposite of excitement and often terminates fatally in a few hours.

And yet so uniformly do they occur in the same localities, and so imperceptibly do the phenomena of the one become merged in those of the other in different cases, that all experienced practitioners acknowledge their origin from simple modifications of the same essential cause. That such cause does act directly on the constituents of the blood as well as the elementary properties of the solid tissues, is evident not only from the analyses which have been made while the disease is in progress, but also from the defective condition of the blood which remains often for a long time after the active febrile symptoms have ceased. This defectiveness is indicated by the altered hue of the skin, the pale or semi-transparent appearance of the pro-labia, and the inordinate muscular weakness, or want of the usual power of endurance; showing at once that the blood corpuscles are so diminished and altered in quality as to be incapable of imparting the natural vigor and activity to the organic actions. It is this same defective con-

Reel, 78-10-5-CONT

dition of the blood remaining after the interruption of the paroxysms of fever by simple anti-periodics that makes intermittents so extremely liable to relapse, unless a judicious plan of treatment is persisted in until it is fully restored. A failure to recognize this fact, and a disposition on the part of both practitioners and patients to regard intermittents as *cured* when the active paroxysms have been arrested, has indeed led to such frequent relapses as to materially shake the confidence of the people in the ability of the profession to *cure* this simple form of fever. If, as I have endeavored to explain in the preceding pages, the true pathology of periodical fevers consists in diminished tonicity* coupled with a morbid development of susceptibility in the solids, and a rapidly increasing impoverishment of the blood, we have a rational explanation of the well known tendencies and sequalæ of that class of diseases. The irritative excitement of the nervous, vascular, and secretive structures, coupled with diminished tonicity in the same parts, and an altered affinity between the solids and fluids, directly disposes first to excited functional action in some organs and rapid congestion in others, and subsequently to more permanent non-inflammatory visceral engorgements and dropsical effusions—I say *non*-inflammatory engorgements, because it is now well known that a very large majority of the visceral obstructions resulting from intermittents and remittents, instead of being the result of inflammatory action, and amenable to antiphlogistic and mercurial remedies, are much more certainly and speedily removed by such means as are calculated to renew and replenish the blood and increase the tonicity or textual vigor of the solids.

And even in those cases where true inflammation does exist in connection with this class of fevers, it is well known that active depletion is not borne to the same extent as in inflammation of the same organs or tissues unconnected with periodical fevers. And not only is active depletion badly borne in such inflammations, but cases are frequently met with in which medicines possessing moderate tonic properties act beneficially.

Hence we are led to infer that inflammation may occur, characterized by pain and tenderness, in parts where the tonicity or

See Appendix Note B.

texture is impaired and the capillary vessels in a relaxed or debilitated state. And it is owing to this element in those pneumonias and other inflammations that occur in malarious districts, which causes them to require a treatment essentially different from the same disease in non-malarious sections of country.

The actual results or post-mortem appearances which have been observed in fatal cases of periodical fever, vary very much, being influenced by the variety and duration of the disease. Simple intermittents so seldom terminate fatally that very few opportunities for making post-mortem examinations have occurred. The malignant or pernicious variety of this disease, however, often terminates fatally in a few hours; and its pathological anatomy has been examined by several physicians in the west and south. Dr. Drake, after alluding to several of these, concludes as follows, viz:—

"To sum up, we may say, that the signs of inflammation were few and uncertain, that *passive* congestions were common; that they occur in the brain and lungs, but still oftener in the abdominal organs; above all in the spleen; but that no organ was always affected, and consequently *that none*, according to these observations, is the invariable and characteristic seat of lesion."

I have had but one opportunity of instituting a post-mortem examination in a well marked case of this disease. In this instance the patient died in the first paroxysm and in little more than twelve hours from the commencement of the attack. The external surface was livid or leaden color, on account of the capillary congestion, but a very careful examination of the brain, spinal cord, and viscera of the chest and abdomen, discovered no marks of disease except simple venous congestion in the membranes of the brain and mesentery. The substance of the brain, the liver, and the spleen were natural in color, size, and consistence, and there were no *visible* lesions sufficient to account for the cessation of life. In such cases the elementary functions of the system are suspended and life becomes extinct before sufficient time has elapsed for any considerable organic changes to occur. Indeed, the whole phenomena, both before and after death, are such as we might suppose would be presented if by some agency or influence the organic susceptibility of the system could be suspended so far that the heart, the lungs, and the capillaries respond very feebly to the presence of their natural stimuli, the blood and the air. The blood becomes retarded in the capillaries, the secreting organs fail to furnish their accustomed secretion, the production of animal heat is greatly diminished or entirely suspended; producing a purplish or leaden color of the lips and sometimes of the whole surface, coldnesss of the extremities, feebleness of pulse, irregular and sometimes sighing respiration, and a sense of great oppression and restless anxiety. If relief is not speedily obtained, life ceases, leaving the veins and more vascular structures more or less engorged with dark blood, but without any other physical evidences of disease. Those who regard the nervous system as the primary seat of morbid action in fevers attribute the death in such cases to a suspension of innervation. Thus, says Dr. Drake, "in several cases, very few traces of disease were found. The *patients died from nervous depression.*"

"The phenomena of *Congestive fever*," says Dr. Condie, "are evidently the result of *defective innervation*, and an impeded action of the heart and lungs; the blood imperfectly decarbonized accumulates in the internal organs and thus prevents the full and regular exercise of their functions. The impression of the morbific causes by which the disease is produced is, in all probability, made *primarily* on the *nervous centre*, and by depressing the energy of its action gives rise to all the other phenomena which characterize this form of fever." It would have been more satisfactory if the advocates of the neurological origin of fevers, had stated more definitely what they mean by "*nervous depression*" and the "*nervous centre.*" Is this *nervous depression* a paralysis or suspension of the functions of a part or of all the nerve matter in the system? Where is the *nervous centre*, of Dr. Condie? Is it the centre of the cerebro-spinal system—the medulla oblongata, or is it the central ganglia of the sympathetic? And what are the phenomena, either during life or after death, which show that the nerve tissues are any earlier or more deeply involved, than the vascular, the visceral, or the muscular? Certainly, so far as post-mortem examinations have been recorded they show much more frequent and decided marks of disease in the viscera than in any part of the nervous system. And the symptoms during life are far from being analogous to those which accompany any well known disease or injury of the brain, medulla oblongata, or spinal cord. The phenomena which accompany inflammation, congestion, concussion, and compression of the cerebro-spinal centres have all been observed and recorded, but we seek among them in vain for symptoms closely analogous to those which mark an attack of fever, whether mild or malignant. The particular pathological condition claimed is that of "nervous depression;" by which I suppose we are to understand that the functions of the "nervous centre" are diminished or suspended. But a diminution or suspension of action in any part of the cerebro-spinal system is well known to produce corresponding impairment or suspension either of the mental faculties or of the sensitive and motor powers of some portion of the physical system. Yet paralysis, whether partial or complete, is nowhere enumerated among the symptoms of pernicious fever, and though dulness of intellect or moderate delirium is sometimes present it is by no means a constant symptom. In the summer of 1851, while the cholera was prevailing as an epidemic in this city, the following case came under my observation, viz: Mr. A., a Norwegian by birth, aged 20 years, sanguine temperament, and good constitution, ate his breakfast as usual in the morning and went to his work as a day laborer. He was soon seized with a feeling of prostration which rendered him unable to continue his work. His companions gave him a little brandy and water, after which he walked home and sent for his physician. Before my arrival he had went to the privy, had one pretty copious watery evacuation from his bowels, returned into the house, sat in his chair, smoked a pipe of tobacco and went to bed. Arriving soon after, I found the aspect of the countenance dull and shrunken, the lips and nails purplish, the skin dingy, and when pressed upon, the blood returned slowly through the capillaries, the extremities very cold, pulse 110, small and easily compressed, impulse of the heart feeble, respiration slow, tongue moist with a thin whitish fur, much craving for cold drink, a sense of great oppression or sinking at the epigastrium, and slight cramps in the muscles of the legs. There was no vomiting, no disturbance of the intellectual faculties, and no apparent loss of either sensation or motion. In this case the remedies prescribed had no visible effect whatever; on the contrary, the purple color of the lips and nails, the lividity and coldness of the surface and tongue, the feebleness of the pulse, and the sense of sinking or oppression rapidly increased until the capillary circulation and pulse at the wrist were entirely suspended, and he died within eight hours after the commencement of the attack. After the first visit there were no discharges either from the stomach, bowels, or bladder; the abdomen was not distended, and the mental faculties with the sensitive and motor functions remained, with no other evidence of disturbance than a debility or indifference which a rapidly failing circulation would necessarily induce, until a few minutes of the fatal termination. I have seen one or two similar cases during each season that the cholera has prevailed here in an epidemic form; and the most careful post-mortem examination can detect no other evidence of disease than the livid and shrunken state of the surface, and the general accumulation of dark fluid, or only partially coagulable, blood in the veins and capillaries. So far are the phenomena during life or after death from indicating an early and profound lesion of the nervous centre, that while watching anxiously by the bedside and seeing the capillary circulation, the secretions, and the animal heat all rapidly diminish until the pulse could no longer be felt at the wrist, the skin, lips and tongue livid and cold as death, and yet the brain continuing to manifest the mental faculties, the nervous cords to transmit the mandates of the will, with a continuance of ordinary sensation and the power of deglutition, I have been compelled to think that of all the important organs in the system, the nervous centres were the least disturbed and the last to be overwhelmed. True, these cases were regarded as cholera rather than pernicious fever; but their similarity to the latter makes them interesting as points for comparison. But it will be said that the failure of the capillary circulation, the suppressed secretions, the feebleness of the heart's action, and the diminution of animal heat, *are the* evidences of *nervous depression*—that the presence of these symptoms constitutes sufficient evidence of a profound lesion of the nervous centres. This would be true if it were first *proved* that capillary circulation, secretion, and animal heat, were wholly controlled and maintained by the agency of the nervous system. That the nervous system exerts some influence over these functions, was long since demonstrated by the experiments of Wilson Philip, Sir Benjamin Brodie and others; and the same is shown by the phenomena which accompany every paralyzed limb.

But it has been demonstrated with equal clearness and certainty that such influence, instead of being supreme and all-controlling, is entirely limited and subordinate.

The truth of this will be at once apparent by referring to the experiments of Weber, Dr. J. Reid, and Muller, in reference to the circulation, and the more recent ones of Dr. Dowler, Dr. M. Cl. Bernard, and Dr. Brown-Sequard.

Dr. Carpenter says in regard to this subject, that "the experiments of Muller and others satisfactorily prove that the ordinary action of the nerves does not produce any direct effect upon the capillary circulation."

Dr. Dowler, in his observations on the circulation and temperature of the body, after death from yellow fever and other rapidly fatal diseases, found that in many instances the capillary circulation was continued for some minutes after the heart's action had entirely ceased, filling the veins to such an extent that a puncture gave exit to a free stream of blood projected several inches from the orifice. Again, it is well known that in cold blooded animals the circulation has been seen to continue after both the heart and the nervous centres had been removed or destroyed; and in the development of the embryo, the *rete vasculosa* or first rudiments of circulation commence, independent both of the heart and nervous system. On the other hand, the observations of Mr. Wharton Jones, Mr. Paget, and Dr. J. Reid, show that the circulation of the blood in the capillaries may be retarded or entirely arrested by changing the qualities of the blood, independent of any change in the action of the heart or the nervous centres. The latter found that when the air had been obstructed in its passage through the trachea of a dog until the blood had assumed a veinous hue in the arteries and the inspirations slow and labored, the systemic capillary circulation became almost entirely suspended. In this state he applied the hæmadynamometer to the femoral artery and found the pressure of blood on the walls of that vessel much greater than natural, while in the veins the pressure was proportionably less. On removing the obstruction to respiration and allowing the blood to become again decarbonized the pressure in the artery returned to its normal condition while that in the veins increased. Mr. Jones found similar effects to be produced by directing a stream of carbonic acid against the capillary net work without any interference with the respiratory process. These facts clearly show that the retarded circulation in the capillaries resulted from an altered relation between the vessels and the contained blood, and not from any fault in the action of the heart or central organs. For in Dr. Reid's experiments, though the action of the lungs and heart were both more or less obstructed, yet the latter continued to send the blood to the capillaries until the pressure in the arterial trunks became much greater than natural, while in the venous trunks it was equally diminished, thereby showing that the obstruction was necessarily in the intervening, systemic, capillaries. Now, if we admit the truth of the proposition, that all organized matter is endowed with a *susceptibility* to impressions without regard to nerve influence, and consequently that the capillaries, secreting cells, and all the elementary structures possess such susceptibility, we can readily account for the results of the experiments just alluded to, as well as many important pathological phenomena. For instance, when the blood was so changed by being deprived of its oxygen, or by being overcharged with carbonic acid, that it was incapable of maintaining the inherent susceptibility of the tissues, the affinity between it and the capillaries ceased; and in the same proportion the passage of blood through them became retarded or entirely suspended. If it is thus certain that there is something beyond mere innervation or the integrity of the nervous system, necessary to establish and maintain the circulation, the proof is even more explicit in regard to the production of animal heat. For, while the experiments of Brodie and the ordinary phenomena of paralyzed limbs, seem to show that a withdrawal of the nervous influence causes a diminution of temperature, there are not wanting many facts of a directly opposite character.

Thus Sir B. Brodie himself relates a case in which a severe injury in the lower part of the cervical region completely paralyzed all the parts below, and yet the heat of the body, as shown by a thermometer placed in the groin, was 111o, Fahrenheit. Dr. Dunglisson relates other cases of the same kind; and M. Cl. Bernard, in a communication in the *Gazette Medicale*, during the past year, states that division of the trunk which unites the sympathetic ganglia in the neck uniformly gives rise to an increase of temperature in the corresponding side of the face, often amounting to 7° or 8°, F.; and that removal of the superior cervical ganglion produces the same effect. The elevation of temperature thus induced is not temporary or dependent on the occurrence of inflammation, but often continues many months. Neither is it prevented by dividing in addition to the sympathetic, the branches of the spinal nerves distributed on the same side of the face. By referring to the *Medical Examiner* for August, 1852, we find the same statements made by Dr. Brown-Sequard, who attributes the increase of temperature to a dilitation of the vessels in the paralyzed part. He further states that if a galvanic current be transmitted through the upper portion of the divided sympathetic nerve, it soon causes a contraction of the capillary vessels of the corresponding side of the face, and with such contraction a reduction of the temperature to the normal standard. From all these facts, it would seem that the nerves exercise only a *limited* influence over the capillary circulation, their injury or destruction in some cases diminishing and in others increasing the circulation of blood in the parts on which they are distributed; and that the temperature increases or diminishes in direct correspondence with the supply of blood. We cannot, therefore, in the present state of our knowledge rightfully claim that the failure of temperature, of capillary circulation, and of secretion which takes place so rapidly in severe cases of pernicious fever, cholera, &c., is merely the consequence of "*nervous depression*."

Such a claim is not merely hypothetical, but it fails to afford a satisfactory explanation of the phenomena presented either during life or after death. For, if it is true that in the most malignant and rapidly fatal form of periodical fever there are less traces of disease in the nervous structures than in the vascular and visceral, the difference is still greater in the milder and more protracted cases. For though intermittent fever in its ordinary form seldom terminates fatally during the immediate progress of the disease, yet it induces changes in the system that are easily observed and which every now and then prove ultimately fatal. These changes instead of being most prominent in the nervous centres, as we should expect if they constituted the primary seat of morbid action, are universally noticed most prominently in the blood, the nutritive processes, or the viscera of the abdomen; and frequently in all these at the same time. I have seen no sequence of disease more uniform than the diminution of red-corpuscles in the blood, and the loss of tone in the solid tissues, in all cases of protracted intermittents. In some instances this change in the blood and tissues generally is sufficient to induce dropsy and death without any visceral enlargements or mechanical obstructions to the circulation in any part of the system. An analysis of the blood in an extreme case of this kind has already been given in this essay; and I have another now under treatment in the Hospital.

In remittent fever the predominant morbid appearances in the blood and the viscera of the abdomen over those in the cerebro-spinal and ganglionic nervous system, is equally well marked. A few years since, Dr. Stewardson, in the Pennsylvania hospital, and Dr. Swett, in the New York hospital, undertook a very careful examination of several cases of remittent fever occurring in those institutions. Dr. Stewardson examined the brain in five cases, and after detailing the appearances remarks as follows, viz: "The above alterations are similar to those found in other acute diseases, and must be regarded as slight and comparatively unimportant, if we except the individual in whom there was large bloody effusion in the ventricles." Dr. Swett also examined the brain in five cases, and reports it for the most part *healthy*, although some of the patients had delirium and coma during life. The viscera of the chest were examined by Dr. Stewardson, in six cases. He found unequivocal evidences of inflammation of the lungs in only one case; and in another they were "highly congested, their color throughout nearly their whole extent being very dark, almost black, and the tissue but slightly crepitant, though not granulated or very easily penetrated." The heart he found "flabby" in three of the six cases examined, and in two there was diminished consistence. "In the same three cases its lining membrane was reddish brown, deep red or violet; in two of these, the coloring being deepest on the right side and in the neighborhood of the valves, and extending into the pulmonary artery and aorta." Dr. Swett, says the "heart was either natural or flabby and softened; and in two cases the lungs showed signs of pneumonia." In the abdominal cavity the chief changes were noticed in the liver, duodenum, and spleen. In regard to the first, Dr. Stewardson says it was "enlarged in three cases, and in one of them to a great degree; in the others it was of natural or moderate size. The consistence of the organ appears to have been generally diminished, being flabby, or softened, or both, in four cases, a little soft in the fifth, and moderately firm though still readily penetrated with the finger in a sixth; in the seventh the consistence is not mentioned." The color in every instance was changed to a bronzed or slate color externally, and olive within. In the five cases examined by Dr. Swett, the condition of the liver corresponded very closely with those of Dr. Stewardson, both as to consistence and color. The latter regards the change of color as peculiar and characteristic of periodical fever. Dr. Swett attributes it to a change in the coloring matter of the bile. Dr. Drake says; "My own occasional autopsies, have afforded results, which correspond very well with those which have been detailed; but I confess that my attention was not attracted to the *peculiar* color of the liver."

In November, 1852, a patient died in the Illinois General Hospital, who according to her own account had been attacked several weeks previous with remittent fever. When she was brought to the hospital, dysentery had supervened, and in a few days more she became almost universally edematous; the effusion being greatest in the lower extremities and abdomen. After death the liver was found enormously enlarged, exactly corresponding in color with those described by Dr. Stewardson, and considerably softened in its texture. On examining it under the microscope it was found to be very copiously infiltrated with fat globules; the latter evidently making up the greater part of the abnormal bulk. The patient was reported to have been addicted to the intemperate use of alcoholic liquors; but the evidence in relation to this was not very reliable. In this case, the spleen was also enlarged but very little altered in texture. In almost all the cases reported, the spleen has been enlarged, engorged with dark blood, and in most of them, softened. In commenting on the nature of the foregoing changes in the various organs, Dr. Drake, in his Systematic Treatise, page 831, very judiciously remarks: "But the admitted ravages of inflammation are neither constant nor striking:—not sufficient, I think, in most instances, to account for the death of the patient; unless we include among them all cases of congestion and softening; which would certainly be gratuitous. *Passive Hyperæmia* is an unquestionable pathological fact; and *fever softens every tissue* of the body. To the latter type of morbid action, we may refer the soft and flabby state of the heart, not less than of the liver, spleen, and mucous membrane of the stomach and duodenum. * * * * The soft and pulpy state of the mucous membrane, with but little appearance of hyperæmia, is doubtless, of a febrile, rather than phlogistic origin."

If it is true, as asserted by Dr. Drake, that fever "softens every tissue in the body;" indeed, if the actual post-mortem phenomena afford any indication concerning the specific nature of the preceding disease, then certainly the impoverished blood and softened textures resulting from protracted and fatal intermittents and remittents strongly sustain the position that one of the primary elements in the pathology of those fevers is a diminution of the *tone* or texture, or in other words, an alteration of the affinity between the elementary particles of which the tissues and the blood are composed. And it is this, coupled generally with an exaltation of the organic susceptibily or irritability, and involving every tissue in which the blood circulates, whether it be nervous, vascular, muscular, or cellular, which constitutes the primary and essential pathology of this class of fevers.

Let us next inquire into the nature of those morbid appearances which are most common in fatal cases of the continued type of fever. Under this head I shall include both the *Typhus* and *Typhoid* of modern writers. Perhaps no subject has been examined with greater care than the morbid anatomy of continued fever. Its almost universal prevalence, its fatality, and the controversy concerning its varieties, have led to more numerous and minute post-mortem examinations, than have been made in reference to almost any other disease. On this point the writings of Louis, Chomel, Jackson, Gerhard, Jenner, and Upham are particularly full and satisfactory in their details. But their facts have been so extensively copied into all the more recent works on the general subject of fever, that they are within the reach of every industrious student of the profession. Hence it is only necessary for my present purpose to detail so many of these appearances as will serve to indicate their extent and nature.

Perhaps no part of the physical system is more constantly altered from the normal condition in all the varieties of continued fever than the blood.

Chomel examined the blood in the heart and aorta of thirty fatal cases of typoid fever, and found " small scanty fibrinous concretions in six, dark coagula in nine, and dark fluid blood in fifteen." Andral and Gavarret made many analyses of blood from typhoid fever patients and generally found the fibrine diminished in direct proportion to the severity of the disease. In no case did they find this constituent increased. Dr. Gerhard describes the blood found in the heart and larger vessels after death from *typhus* fever as uncoagulated, thick in consistence and very dark color. Dr. Jenner says, " the fluid condition of the blood generally, was observed in about equal proportions in the subjects dead from typhoid and typhus fevers; but it was far more profoundly diseased, i. e., it deviated far more from its healthy condition in the cases of typhus than in those of typhoid fever." Dr. Upham noted particularly the condition of the blood contained in the heart and larger vessels, after death from " maculated typhus or ship fever" ; and he uniformly describes it as " thin, dark, and oily"—or "fluid, dark and sizy, its clot soft and readily broken down." My own examinations of the blood in continued fever have chiefly been made during the life of the patient; and in no well marked case have I found it presenting the same appearances as in health. The appearances have pretty uniformly corresponded with those particularly described in case third, the analysis of which has already been given in detail.

As remarked in connection with that case, I have uniformly found far less alteration in the relative proportion of the constituents of the blood in continued than in periodical fevers, but a much greater alteration of the *qualities* of such constituents. I have not even found the fibrine as deficient as represented by Andral and Gavarret, but it always coagulates much more slowly and forms a much less firm clot than in health. The red globules too, are much more irregular in size and darker color both in the arteries and veins than natural. These changes in the appearance and qualities of the blood, seem to bear a pretty direct ratio, in degree, to the severity and prostration characterizing the disease. Next to the blood, the organs most uniformly affected in continued fever are the mucous membrane of the alimentary canal and the spleen. In the more severe and rapidly fatal cases and those usually styled typhus, the mucous membrane of the stomach and duodenum is in many cases softened, especially near the great curvature of the stomach, and in some cases ecchymosed and in others paler than natural. The same appearances are sometimes seen in the mucous membrane of the ilium, but ulcerations are comparatively rare. In the more protracted cases, and those occurring in subjects between the ages of 18 and 35 years, softening of the mucous coat of the stomach is less frequent, but the aggregated glands of Peyer in the small intestines are very generally injected, softened, and ulcerated. Sometimes the ulcerations are very extensive, occasionally perforating all the coats and inducing a rapidly fatal peritonitis. But the extent of these ulcerations are not found to bear any uniform relation to the severity of the disease; many fatal cases occurring with but slight affection of the glands or none at all.

With ulcerations in the ilium we almost always find the glands in the corresponding portions of the mesentery enlarged, often softened, and sometimes the peritoneum covering them bearing marks of inflammation. In one morbid specimen now in my possession in which the glands and mucous membrane of the ilium were extensively ulcerated several of the glands in the annexed portion of the mesentery were enlarged and their substance reduced to a semi-fluid consistence; and in its recent state two or three spots on the peritoneum were injected with blood and covered with a layer of coagulable lymph or false membrane. In the same subject the membrane lining the larger bronchial tubes was injected with blood, dark red, and softer than natural. In another specimen which was taken from a patient that several weeks previous had labored under some pulmonary disease, but who subsequently died with typhoid symptoms, the mucous membrane of the ilium is extensively injected, in some portions intensely red, in others dark brown, and throughout studded with small ulcerations both in the aggregated and solitary glands. But in this case the mesenteric glands instead of showing signs of inflammation or softening, are very much enlarged and filled with hard calcareous matter. In almost all the cases of continued fever terminating fatally that have been examined, more or less changes from a normal condition have been noticed in the spleen. In many cases it is enlarged and darker color than natural; and in nearly all it is described as filled with dark fluid blood, somewhat softened in texture, and sometimes so broken down that it tares as readily as an ordinary clot of blood. The liver is much less frequently diseased than the spleen. In the majority of cases it is reported healthy or only slightly altered; but sometimes it is enlarged or softened, or both. The lungs have been found in an abnormal condition almost as frequently as the spleen. The most constant morbid appearance is that of passive engorgement with dark blood, almost always in the lower and posterior, or dependent parts of the organs. So great is this engorgement in some cases that the affected part more resembles an enlarged and softened spleen than the natural tissue of the lungs. In those cases which have run a protracted course, especially in the middle period of life, portions of the lungs are often found consolidated and sometimes granular, as if a low grade of pneumonic inflammation had existed. The muscular structure of the heart is often found more flabby or softer than natural and its inner surface deeply stained with dark colored blood. Of all the important organs named the brain presents the least constant and serious marks of disease. Chomel carefully examined the brains of 38 fatal cases; and in 15 he found no morbid appearances whatever; in 12 from one to two drachms of serous fluid were found in the ventricles; in 7 there was some effusion of a similar fluid in the meshes of the pia mater; in 6 a slight diminution of the consistence of the cerebral substance; in five there was a slightly injected appearance of the substance of the brain. Louis examined 48 cases; Dr. J. Reid more than 150; Jenner 35; and in all, the results correspond very well with those detailed by Chomel. Generally the membranes of the brain are more or less congested with dark fluid blood, and in a large majority of cases this is the only morbid appearance, even where delirium and coma have been very prominent symptoms during life. So true is this, that Dr. Bartlett observes, "there is no ascertained relation between the cerebral symptoms during life, and the pathological conditions of the brain and its membranes, appreciable after death.

Delirium and somnolence are found to have occurred as frequently, and to have been as strongly marked, in patients whose brains presented no changes, or only very slight ones, after death, as in those of an opposite character." See Bartlett on Fevers, page 90, third edition.

Dr. Watson also, when speaking of the cause of coma which is a frequent symptom, especially in fatal cases of continued fever, says: "Physicians have diligently searched for it by examining the dead brain. I cannot tell you how often I have looked, and looked in vain, for some palpable disorganization, or some effusion implying pressure. All who are familiar with the dead-house of a hospital are aware that this fruitless search for some physical explanation of the comatose state, after death by fever, is of very common occurrence." See Watson's Practice, p. 942, 3d Ed.

(*Continued next month.*)

